# Domestic Correspondence

**Published:** 1884-04

**Authors:** 


### Domestic Correspondence.

TO THE EDITORS OF THE CHICAGO MEDICAL JOURNAL AND EXAMINER.

*Gentlemen:* Recently I was accorded space in your journal for a statement in relation to the early history of the Cook County Insane Asylum and Poor House, in response to an article therein from the pen of Dr. Clevenger, of the present medical staff of this institution, in which the same was distorted and an injustice accordingly done to those formerly connected therewith as officials, together with myself as physician in charge.

Another article has appeared in your January number, which compels me to crave a further indulgence in your columns in the interests of fairness and of the profession. Reference is had to the case reported by A. W. Hagenbach, M.D., Superintendent of the Cook County Infirmary, under the head of "Ligation of Femoral and External Iliac Arteries," in which I am referred to as having made the primary amputation.

Without courting controversy with any of the students connected with the medical staff of this institution, a reference to this case on my part becomes excusable, if not, in fact, demanded. That I should be singled out again, and my practice and skill be made the subject of invidious comparison with that of the parlo scribes of a public institution is doubtless due to the proximity of my location as a practitioner. Members of the profession more distantly located are to be congratulated in escaping the annoyance to which I am being subjected. Situated near the point of convergence of several railroads, accidents necessitating amputations are of frequent occurrence, many of the patients proving to belong to the class known as "tramps" or paupers. The result has been a contribution to me of a moderate line of surgical practice, by no means lucrative, and entirely unsolicited. Under such circumstances, it is most respectfully submitted that the amenities of the profession should not allow the operator to be deprived of the only compensation received in such cases, viz.: a fair recognition and treatment of his services performed.

Only a brief reference need be made to the history of the case in question as given by Dr. H. Its importance can be weighed with prescription scales.

According to the statement made by him, the patient in the case was received from me, after the primary operation, on "October 22, 1882." From this date to "October 26, 1882," no account of the condition of the patient is historically vouchsafed in the report. From thence to "November 9, 1882," covering a period of *fourteen* days, the profession is left without a word as to the condition and treatment of the patient, a degree of incompleteness unaccountable in the make up of a reported case designed to afford the least instruction to the profession. Under a course of subsequent treatment such as would be inferred from such a history, what reader, professional or otherwise, would make the slightest investment in the notion conveyed by the report as a whole, that the amputation as performed by me was such as that reasonable treatment and care thereafter would not have restored the patient? But, continues Dr. H., at this time "both bones protruded, and there was found a *large bundle* of silk ligatures cut close to the arteries, ligated at bottom of wound, etc." Allowing that there were but two arteries ligated, as would be usual in such cases, about what sized "bundle" would he claim "two silk ligatures cut close to the arteries ligated" makes? The most visionary stretch of fancy should not lead to such a reckless misapplication of terms. No surgeon has as yet been known to exhaust his entire stock of ligatures upon one patient, and such an event is not likely to occur.

A reference to my record of the case shows to a certainty that the patient was received by me, and the amputation performed immediately after the accident, on *November* 1, 1882, while claimed to have been under treatment at the infirmary from

"October 22, 1882," a period of *eleven* days prior to the accident! If the claim be true, then the patient must have been first received as a guest at the Poor House, and the doctor must have got his accompanying "bundle" as a pauper, confused with his subsequent "sutures" as a patient.

There is no disposition, however, on my part, to take any exceptions to the treatment bestowed upon the patient by Dr. H. covering that period prior to the accident and amputation. It was doubtless the best part of it.

In cases of this kind that may arise hereafter, after an amputation has been performed by me and a transfer of the patient to any public infirmary for treatment and care, I shall be disposed to claim a return of the patient if any difficulty is encountered thereat in effecting a permanent cure and restoration, but it must be done in season, and not after any lengthy experimentation upon the patient has been practiced.

In giving my version of the case reported, I beg leave simply to submit the following affidavits, which furnish their own explanation, and with the single observation that the object of this communication will be accomplished if my professional reputation shall be removed from under not a *cloud*, but a *shadow*, and medical literature hereafter be promulgated with a larger degree of fairness and accuracy. D. B. FONDA, M.D.

Dated at Jefferson, this 5th day of February, 1884.

STATE OF ILLINOIS }
COOK COUNTY. } *ss.*

I, David B. Fonda, being duly sworn, on oath say: That on November 1, 1882, at his office in Jefferson, Cook County, Illinois, he, as a physician and surgeon, performed an amputation of the left leg of one J. C. McMullen, being the same person thereafter committed to the charge of Dr. A. W. Hagenbach, Physician in charge of the then Cook County Poor-House, and the one made mention of by him in the January number of the CHICAGO MEDICAL JOURNAL AND EXAMINER; that the operation was performed by him in person and in presence of, and with the assistance of, Drs. C. W. Mercereau and W. Snyder, of Jefferson, and Mr. Fred. T. Franks, a student of medicine of Chicago, and on account of the following injury sustained by attempting to alight from a moving freight train, on the Chicago and Northwestern Railway; at Plank Road Station, viz.: Compound comminuted fracture of the left tibia and fibula, necessitating the same, and in following manner, to-wit: The amputation was made in the upper portion of the middle third of the leg, with long posterior flap; that each of the arteries were separately ligated at the hands of Dr. Mercereau with silk ligatures, ONE strand of which, in each instance, was cut short at the point of ligation, the other carried external to the wound and fastened externally by an adhesive strap binding them to the anterior part of the leg, and as an auxiliary measure of protection; that the flaps were brought in apposition by five (only) interrupted sutures, and secured by adhesive strips and bandage; that the tourniquet was adjusted in manner to compress the popliteal artery, and left in position, with instructions to the person removing the patient to tighten the same in case bleeding should occur during process of removal, and for the purpose generally of guarding against accident from a possible secondary hæmorrhage: that a note from him to Dr. Hagenbach accompaned the patient, setting forth the above facts, and with a request that he take charge of and return the tourniquet after its use.

D. B. FONDA, M.D.

Subscribed and sworn to before me, a notary public, this 5th day of February. 1884. ISAAC N. HUESTIS.

[Notarial Seal.]

STATE OF ILLINOIS, }
COOK COUNTY. } *ss.*

I, Fred. T. Franks, being duly sworn, on oath say: That on November 1, 1882, I was present at, and assisted Dr. D. B. Fonda to perform, the amputation described in his affidavit, and that I do know of my own knowledge that it was done on the day, and in every particnlar in manner and form as described and set forth in his affidavit. FRED. T. FRANKS.

Subscribed and sworn to before me, a notary public, this 5th day of February, 1884. ISAAC N. HUESTIS.

[Notarial Seal.]

STATE OF ILLINOIS, } ss.

COOK COUNTY. }

I, C. W. Mercereau, being duly sworn, on oath say: That on November 1, 1882, I was present at and assisted Dr. D. B. Fonda in performing the amputation described in his affidavit; that I ligated the arteries in manner as described by Dr. Fonda, and that the amputation was made the day, and in manner as therein described. C. W. MERCEREAU.

Subscribed and sworn to before me, a notary public, this 5th day of February, 1884. ISAAC N. HUESTIS.

[Notarial Seal.]

---

BURLINGTON, IOWA, March 12, 1884.

MESSRS. EDITORS:—I take the liberty of communicating to you a fact corroborating a statement made by Dr. Lagorio at the meeting of the Chicago Medical Society on the 18th inst.

Two years ago, while visiting a patient a short distance in the country, my attention was called to a newly born blind calf. It was perfect in every way, with the single exception that it had no eyes and no place for them. There was no depression in the skull where the orbits should have been.

I have also in my possession the head of a pig, with a single large eye in the center of the forehead. There is a projection over it, of a cylindrical portion of the integument resembling the penis of a boy. There is, however, no aperture in the latter.

I should have mentioned that the calf was suckled well, and the farmer sold it to a butcher, who had served it out as veal before I could secure the specimen.

Yours truly,

G. R. HENRY, M.D.

